# The relationship between indicators of socioeconomic status and cesarean section in public hospitals

**DOI:** 10.1590/S1518-8787.2017051006134

**Published:** 2017-02-21

**Authors:** Alexandre Faisal-Cury, Paulo Rossi Menezes, Julieta Quayle, Kely Santiago, Alicia Matijasevich

**Affiliations:** I Departamento de Medicina Preventiva. Faculdade de Medicina. Universidade de São Paulo. São Paulo, SP, Brasil

**Keywords:** Cesarean Section, Risk Factors, Socioeconomic Factors, Hospitals, Public, Obstetrics

## Abstract

**OBJECTIVE:**

To assess the relationship between indicators of socioeconomic status and cesarean section in public hospitals that adopt standardized protocols of obstetrical care.

**METHODS:**

This was a prospective cohort study conducted between May 2005 and January 2006 with 831 pregnant women recruited from 10 public primary care clinics in São Paulo, Brazil. Demographic and clinical characteristics were collected during pregnancy. The three main exposures were schooling, monthly family income *per capita*, and residential crowding. The main outcome was cesarean section at three public hospitals located in the area. Crude and adjusted risk ratios (RR), with 95% confidence intervals were calculated using Poisson regression with robust variance. We examined the effects of each exposure variable on cesarean section accounting for potential confounders by using four different models: crude, adjusted by mother’s characteristics, by obstetrical complications, and by the other two indicators of socioeconomic status.

**RESULTS:**

Among the 757 deliveries performed in the public hospitals, 215 (28.4%) were by cesarean section. In the bivariate analysis, cesarean section was associated with higher family income *per capita*, higher education, lower residential crowding, pregnancy planning, white skin color, having a partner, and advanced maternal age. In the multivariate analysis, after adjustment for covariates, none of the socioeconomic status variables remained associated with cesarean section.

**CONCLUSIONS:**

In this group, the chance of women undergoing cesarean section was not associated with indicators of socioeconomic status only, but was defined in accordance with major obstetric and clinical conditions.

## INTRODUCTION

Cesarean section is a surgical intervention performed in situations of significant risk to mother or fetus during pregnancy or labor. However, this procedure has been associated with higher rates of maternal and infant morbidity and mortality^28^. A large cross-sectional study conducted between 2004 and 2008 by the World Health Organization (WHO) across 24 countries showed that cesarean section is associated with increased risk of severe maternal outcomes such as death, admission to intensive care unit, blood transfusion, and hysterectomy^27^. Moreover, there is strong evidence of the negative impacts of cesarean on the reproductive future of women^20^. Cesarean rates are above 30.0% in many countries such as Portugal, Mexico, Chile, and Italy^23^, well above the 15.0% recommended by WHO^29^.

In Brazil, from 1997 to 2011, the proportion of caesarean sections among the total number of births raised from 39.8% to 53.7%^a^. The increase was partly associated with the higher rates of cesarean deliveries in the public sector. For example, in 2004, among births in the private healthcare sector, 79.7% (246.264) were by cesarean section. For the same period, the Brazilian Unified Health System (SUS) reported a 27.5% cesarean delivery rate^b^.

Latest data show a high proportion of cesarean deliveries in 2011, especially in the Midwest, the South and Southeast regions of the Country. In public establishments, most births are via vaginal delivery (61.7%), but the proportion of cesarean (38.3%) is still quite high^c^.

The increasing rates of cesarean delivery cannot be explained only by medical indications. Maternal requests and physicians performing unnecessary surgeries (without clear clinical or obstetric indication) are other possible reasons for this increase in the private sector^7^. The current rate of cesarean section varies from 1.0% to 48.0% in the public sector and 60.0% in the private sector^17^. The variation is significant among countries, ranging from 2.0% in Canada^14^ to 80.0% in Brazil^24^. However, unlike the private sector standards, the practice of cesarean delivery due to maternal request in the public service is unusual in Brazil, with a strong support for natural childbirth. In addition, in public teaching maternities, obstetric protocols are well defined and cesarean section only occurs associated with diagnoses of maternal or fetal pathology or special circumstances during delivery.

Other sociodemographic factors associated with cesarean section are ethnicity^24^ ^,^ ^26^, income or education^3^ ^-^ ^5^ ^,^ ^11^ ^,^ ^18^ ^,^ ^24^, and living in an urban or metropolitan area^24^. Several studies have highlighted the important question of social inequality in the distribution of cesarean rates. Thus, women with low socioeconomic status and therefore greater risk of obstetric complications are, paradoxically, less likely to undergo cesarean delivery, when compared with women with low obstetric risk and high income^13^. Sakae et al.^25^ conducted a cross-sectional study at the University Hospital of Florianópolis, SC, Southern Brazil, from 2001 to 2005, and concluded that women in poor socioeconomic conditions had less access to cesarean section in comparison with those women with low obstetric risk and high economic status^25^. Béhague et al.^4^ emphasize that many women’s preference for cesarean section is linked to the misconception that this type of delivery represents the highest quality of obstetric care.

The association between different indicators of socioeconomic status and cesarean delivery in public services is controversial. One case-control study^6^, in two public institutions in Rio de Janeiro, Southeastern Brazil, found no association between women’s years of education and the occurrence of cesarean section, but the risk of cesarean delivery was 3.4 times higher among women who requested a cesarean section. In a cross-sectional study, Mendoza-Sassi et al.^19^ have compared the rates and associated factors of caesarean section in public and private services in Rio Grande do Sul, Southern Brazil, and found that higher income and education were related to cesarean deliveries. The homogeneity of patients’ socioeconomic profile in the public service and the characteristics of the patient-physician relationship, in the private service, were considered important explanations for the lack of association between socioeconomic indicators and cesarean section^1^ ^,^ ^6^ ^,^ ^12^. However, the obstetrical care model, whether or not based on standardized protocols of conduct, is usually less valued to explain this lack of association.

In São Paulo, Southeastern Brazil, the public obstetrics services are heterogeneous, regarding not only the population served and the size of the catchment area, but also the composition of the obstetrics team, the participation of midwives during the labor, the presence of someone supporting the mother, and regular use of analgesia. Although some national policies promote normal childbirth^d^, not all public hospitals adopt obstetric protocols. Most frequently, the obstetrician on call decides about the type of delivery. Similarly, the practice of asking for a second opinion to decide for a cesarean section is not well accepted by Brazilian doctors^21^, although it was proved to be, at least partly, effective in reducing operative deliveries^1^. On the other hand, in the teaching maternities, humanized childbirth is valued by the obstetric multidisciplinary team (nurse, anesthetist, midwife, social worker, psychologist, obstetrician) and they usually adopt standardized obstetric protocols aiming at evidence-based obstetric care. Thus, the high rates of caesarean section in Brazil reflect the participation of many factors, including medical, sociocultural, institutional, financial, and legal variables^10^. Several efforts have been proposed to reduce the rate of cesarean section in the Country, and fighting social inequality associated with this surgical procedure is only one of them. Public hospitals that have adopted standardized obstetric management, in theory, work in this direction, since they offer to all pregnant women the same medical care regardless of social factors.

The aim of this study was to evaluate the relationship between indicators of socioeconomic status during pregnancy and cesarean delivery in public hospitals that adopt standardized protocols of obstetric care.

## METHODS

### Study Design

This was a prospective cohort study conducted between May 2005 and January 2006 with low-income pregnant women recruited from public primary care clinics in São Paulo, Southeastern Brazil. The study area comprised a heterogeneous population of approximately 250,000 inhabitants, with high, middle and low-income people living close to each other, located in the western region of the city of São Paulo. Private health care is usually only accessible for women from the middle and upper middle classes. Public primary care clinics offer free antenatal care for all women living in their catchment areas. Prenatal appointments are offered on a regular basis, usually once a month, starting as soon as the pregnant woman seeks antenatal care for the first time. Antenatal coverage is adequate, with most pregnant women having between four and seven prenatal appointments. Most low-risk pregnant women are followed up in these public primary care clinics. High-risk pregnancies associated with complications are usually referred to a hospital early during antenatal care. There are two public secondary teaching hospitals located in the area of the study, which offer obstetric care for low and high-risk pregnancies at the time of delivery. These two hospitals are responsible for some 2,000 deliveries per year. Pregnant women in their 20th to 30th weeks of pregnancy, naturally conceived, aged 16 years or older, with singleton pregnancies, who were attending antenatal care in one of the primary care units of the study area were eligible. Further details of the study sample have been described elsewhere^9^.

### Measurements

#### Participants’ Characteristics

Sociodemographic and clinical characteristics were elicited through a detailed structured questionnaire applied during pregnancy. These factors include mother’s age, skin color, marital status, planning of pregnancy, number of pregnancies, previous miscarriage, and smoking status. Body mass index (BMI) was assessed and participants were classified in three groups: underweight (below 19.9 kg/m^2^), normal weight (20-24.9 kg/m^2^), and overweight or obese (above 25.0 kg/m^2^).

#### Main Exposure Variables

The three main exposure variables used for the assessment of socioeconomic status were: years of education (0-4, 5-8, 9 years or more); monthly family income *per capita* (in USD), defined as the monthly family income divided by the number of adults and children living in the house (0-59, 60-113, 114-810); and residential density, defined as the number of adults and children living in the house divided by the number of rooms in the house (0.1-0.9, 0.91-1.4, 1.5-8.5).

#### Main Outcomes

The main outcome was cesarean section. Obstetric data were extracted from medical charts from the three public hospitals located in the area (Hospital das Clínicas, Hospital Universitário, and Hospital Sara Kubicheck). Hospital das Clínicas and Hospital Universitário are linked to the Faculdade de Medicina of Universidade de São Paulo. They share the same staff and follow strict obstetrical guidelines for prenatal assistance and delivery. All other data (including data about socioeconomic status) were collected from personal interview. Preterm birth was defined as a delivery before completing 38 weeks of gestation. The Capurro Index was used to assess gestational age at delivery. Low birth weight was defined as below 2,500 grams. Newborn weight is evaluated after delivery, routinely in Brazilian maternity facilities. In the city of São Paulo, there is virtually no delivery outside of hospital facilities. Newborns are usually weighed in the obstetric room, with the use of scales, under the supervision of a trained nurse or a pediatrician. Medical data is registered immediately after delivery. A dual “yes-no” classification of obstetric complications was developed. “Yes” was defined by the presence of gestational age less than 37 weeks or weight of newborns under 2,500 grams or five-minute Apgar less than seven. A list of main medical reasons for cesarean delivery was extracted from medical charts and included: fetal distress and presence of meconium stain during labor and delivery, hypertensive disorders (chronic or pregnancy related), breech presentation, and presence of one or more previous cesarean section. The classification of hypertensive disorders (chronic or pregnancy related) was based in the measurement of blood pressure during admission and labor in the pregnant women clinical data. Place of delivery was classified in university public hospital (Hospital Universitário and Hospital das Clínicas) and public hospital (Sara Kubicheck).

## Procedures

Pregnant women who met the inclusion criteria were identified during their antenatal appointments at the primary care units and were invited to participate in the study. Those who agreed signed an informed consent and were interviewed by research trained assistants, in a private room, where they answered the questionnaire on sociodemographic and obstetric history. The main investigator reviewed all obstetric records from the three hospitals. The Ethics Committee of the Faculdade de Medicina of Universidade de São Paulo approved the research project (Process 475-4).

## Statistical Analysis

Descriptive frequencies were summarized, and all variables studied were categorized. Bivariate analyses were used to examine the association between the three main exposure variables (years of education, monthly family income *per capita* and residential density, and cesarean delivery controlling for potential confounding variables. Crude and adjusted risk ratios (RR), with 95% confidence intervals (95%CI), were calculated using Poisson regression with robust variance to examine the associations between socioeconomic variables with cesarean delivery. We examined the effects of each exposure variable on cesarean delivery accounting for potential confounders by using four different models: (i) unadjusted association; (ii) adjusting for model 1 plus mother’s characteristics (mother’s age, skin color, marital status, planning of pregnancy, number of pregnancies, previous miscarriage, smoking status, and BMI) and place of delivery; (iii) adjusting for model 2 plus obstetrical complications; (iv) adjusting for model 3 plus the other two indicators of socioeconomic status. Covariates were identified *a priori* based on previous research on cesarean delivery and socioeconomic factors. To be included as potential confounders, variables had to be associated with socioeconomic factors and cesarean delivery with a p < 0.2. Statistical associations were assessed with likelihood ratio tests. The Pearson correlation test was used to evaluate the correlation among the three indicators of socioeconomic status. Statistical analyses were performed using Stata 11 software.

## RESULTS

Eight hundred and thirty-one pregnant women entered the study. It was not possible to obtain data about delivery in three cases. Five hundred and nine (61.5%) babies were delivered at the Hospital Universitário; 18 (2.2%) at the Hospital das Clínicas, and 230 (27.7%) in the Hospital Sara Kubicheck. We did not include in this analysis the data for 71 deliveries performed in private hospitals and other public hospitals that do not employ standardized obstetric protocols. The 757 pregnant women included were mainly white (44.8%), in a stable relationship (75.1%), with a mean age of 25.0 years (range: 16-44). Two hundred and fifty–nine (34.2%) women were in their first pregnancy. Two hundred and forty-five (32.3%) women had planned their pregnancy, and 174 (22.9%) had a previous abortion. Half of these women (50.0%) had less than nine years of education. Approximately 72.6% of these families had a monthly income of 400 US dollars or less. The average crowding by room in the house was 1.4 (range 0.16 to 8.5), and 360 (47.5%) households had between 1.5 and 8.4 persons per room. Although significant, all correlations between the three indicators of socioeconomic status were not high (correlation between monthly family income *per capita* and years of schooling 0.20, p < 0.001; correlation between monthly family income *per capita* and residential density -0.44, p < 0.001; correlation between years of schooling and residential density -0.22, p < 0.001).

Three hundred and seventeen (41.9%) women were classified as overweight or obese. Six hundred and thirty (83.2%) women were classified as non-smokers. On the other hand, 7.0% of women smoked more than 10 cigarettes per day during pregnancy. Among the total number of deliveries performed, 215 were by cesarean sections, including 144 among 527 (27.3%) in university hospitals and 71 among 230 (30.8%) in the hospital that was not linked to a university (Table 1). The most frequent indications for cesarean complications were the following: hypertension (chronic or pregnancy-specific) (99; 13.0%), presence of meconium (81; 10.7%), elective cesarean section (26; 3.4%), fetal distress (24; 3.1%), breech presentation (7; 0.9%), intrapartum bleeding (12; 1.6%), and iterative cesarean section (10; 1.3%). In 301 (39.7%) women, oxytocin has been used during childbirth. As for the newborn, 120 (15.9%) and 56 (7.4%) were classified as preterm and low birth weight, respectively (Table 2).

**Table 1:** Sociodemographic, socioeconomic, obstetric, and other health-related characteristics of the sample, according to cesarean delivery.

Variable		Cesarean section		p

	N	Yes	%	

		N		
Monthly family income *per capita* (USD)				< 0.02
0-59	246	53	21.5	
60-113	255	69	27.0	
114-810	233	84	36.0	
Education (years)				0.02
0-4	141	30	21.3	
5-8	239	62	26.0	
≥ 9	375	123	32.8	
Crowding				< 0.001
0.1-0.9	211	81	38.4	
0.91-1.4	186	52	27.9	
1.5-8.5	360	82	22.8	
Skin color				0.21
White	339	104	30.7	
Black/Mixed/Other	418	11	26.5	
Marriage status				0.087
Unmarried	188	44	23.4	
Married	569	171	30.0	
Mother’s age				0.02
16-19	160	38	23.7	
20-29	423	114	26.9	
30-44	174	63	36.2	
Previous miscarriage				0.93
No	583	166	28.5	
Yes	174	49	28.1	
Number of pregnancies				0.07
1	259	82	31.6	
2	232	71	30.6	
≥ 3	266	62	23.3	
BMI (kg/m^2^)				0.044
20.0-25.0	388	101	26.0	
15.4-19.9	52	10	19.2	
≥ 25.1	317	104	32.8	
Smoking				0.38
No	630	183	29.0	
Yes	127	32	25.2	
Pregnancy planning				0.049
Unplanned	512	134	26.2	
Planned	245	81	33.1	
Place of delivery				0.32
University Hospital	527	144	27.3	
Public Hospital	230	71	30.8	

BMI: body mass index

**Table 2:** Obstetrical complications according to cesarean delivery.

Variable		Cesarean section		p

	N	Yes	%	

		N		
Oxytocin use during labor				< 0.001
No	456	159	34.9	
Yes	301	56	18.6	
Breech presentation				< 0.001
No	750	208	27.7	
Yes	7	7	100	
Fetal distress				< 0.001
No	733	191	26.0	
Yes	24	24	100	
Meconium stain				< 0.001
No	676	166	24.6	
Yes	81	49	60.5	
Intra-partum bleeding				0.003
No	745	207	27.8	
Yes	12	8	66.7	
One previous cesarean				< 0.001
No	731	189	25.8	
Yes	26	26	100	
Two or more previous cesarean				< 0.001
No	747	205	27.4	
Yes	10	10	100.0	
Preterm				0.48
No	634	184	29.0	
Yes	120	31	25.8	
Low birth weight				0.53
No	699	197	28.2	
Yes	56	18	32.1	
Hypertensive disorders				< 0.001
No	658	170	25.8	
Chronic hypertension	53	20	37.7	
Pre-eclampsia	46	25	54.4	

In the bivariate analysis, regarding social and demographic indicators, the cesarean was associated with higher familiar income *per capita*, higher education, lower residential crowding, pregnancy planning, and advanced maternal age (Table 1). Regarding to obstetric complications, cesarean section was associated with hypertension (chronic or pregnancy-specific), meconium, one or more prior caesarean deliveries, acute and chronic fetal distress, breech presentation, intrapartum bleeding, and use of oxytocin during labor (Table 2).

In the multivariate analysis, after adjustment for maternal characteristics (model 2), the socioeconomic status variables remained associated with cesarean section. However, after adjustment for obstetric complications (model 3) only residential density maintained this association. Finally, after adjusting for the two other remaining socioeconomic status variable (model 4), the association between cesarean section and residential density was no longer significant (Table 3).

**Table 3:** Crude and adjusted associations of socioeconomic variables with cesarean delivery.

Variable	Model 1: unadjusted	Model 2: Model 1 plus mother’s characteristics	Model 3: Model 2 plus obstetric complications	Model 4: Model 3 plus SE variables
			
	RR (95%CI)	RR (95%CI)	RR (95%CI)	RR (95%CI)
Monthly family income *per capita* (USD)	p < 0.002	p = 0.044	p = 0.13	p = 0.55
0-59	1.0	1.0	1.0	1.0
60-113	1.25 (0.91–1.71)	1.15 (0.84–1.56)	1.13 (0.87–1.48)	1.05 (0.79–1.38)
114-810	1.67 (1.25–2.24)	1.45 (1.06–1.99)	1.32 (1.00–1.74)	1.16 (0.86–1.55)
Years of education	p = 0.02	p = 0.031	p = 0.19	p = 0.85
0-4	1.0	1.0	1.0	1.0
5-8	1.22 (0.83–1.79)	1.31 (0.89–1.92)	1.08 (0.77–1.52)	1.14 (0.81–1.62)
≥ 9	1.54 (1.08–2.18)	1.58 (1.10– 2.27)	1.27 (0.93–1.73)	1.24 (0.90–1.71)
Crowding	p = 0.000	p = 0.027	p = 0.016	p = 0.092
0.1-0.9	1.0	1.0	1.0	1.0
0.91-1.4	0.73 (0.54–0.97)	0.77 (0.57–1.03)	0.84 (0.65–1.09)	0.87 (0.67–1.14)
1.5-8.5	0.59 (0.46–0.76)	0.69 (0.52–0.91)	0.70 (0.54–0.89)	0.74 (0.57–0.97)

Monthly family income *per capita* adjustment:

- Model 2: adjusted by model 1 plus marriage status, mother’s age, number of pregnancies, and place of delivery

- Model 3: adjusted by model 2 plus obstetric complications

- Model 4: adjusted by model 3 plus crowding and years of education

Years of education adjustment:

- Model 2: adjusted by model 1 plus marriage status, mother’s age, number of pregnancies and place of delivery

- Model 3: adjusted by model 2 plus obstetric complications

- Model 4: adjusted by model 3 plus crowding and monthly family income *per capita*

Crowding adjustment:

- Model 2: adjusted by model 1 plus marriage status, mother’s age, number of pregnancies and place of delivery

- Model 3: adjusted by model 2 plus obstetric complications

- Model 4: adjusted by model 3 plus years of education and monthly family income *per capita*

## DISCUSSION

Our study shows that, in public hospitals that adopt standardized obstetric protocols, indicators of socioeconomic status are not associated with higher rates of cesarean sections. Therefore, in these obstetrics services, there is no relationship between women’s socioeconomic status and type of delivery, but the chance of the women undergoing cesarean section is determined by clinical and obstetric indications.

In the public hospitals included in this study, the obstetric care involves nursing staff and doctors with whom the mother did not have direct previous contact. The obstetrical management is defined by standard medical criteria and there is little (or no) chance of pregnant women choosing the type of delivery to which they will be submitted. Contrary to Hotimsky et al.^16^, this distance between prenatal care and delivery in the public service can be considered a contributing factor for cesarean sections, because of the lack of information at the time of delivery about the current and previous pregnancy and because of the failure to establish a good relationship between doctor and patient. Although the rate of cesarean delivery in our sample was 30.3%, still a high value according to WHO’s recommendation^29^, it was significantly lower in comparison with the rates of other public and private hospitals in Brazil.

It is difficult to compare the results of our study with those conducted in other countries, in particular with high-income countries, since demographic characteristics and obstetric care models differ significantly^23^. Equity in the obstetric assistance has been a goal for several developed countries, especially those that adopted lower use of technology and a model less centered in the physician-patient relationship^23^. However, there is evidence that equity associated with cesarean delivery is changing in some countries. For example, a Scottish study^8^ examined the association between two socioeconomic indicators (social class and place of residence) and the cesarean section rate in two periods. They found that, between 1980-1981 and 1999-2000, the rate of emergency cesarean was more common in poorer women and those living in deprived regions. In 1999-2000, women with these characteristics were less likely to undergo elective cesarean section in comparison with more affluent women. For the authors, the determinants of this change are not clearly documented in the medical records and their ongoing investigation is essential to ensure equity in obstetric care^8^.

In Brazil, studies differ about the higher incidence of cesarean delivery associated with indicators of socioeconomic status in public services. A cross-sectional study conducted at a university hospital in Santa Catarina presented data from 2,905 deliveries, evaluating the medical and non-medical factors associated with the increase of cesarean section rates between 2002 (28.4%) and 2004 (36.7%). Higher maternal education, time of delivery, presence of pathology, and increased frequency of prenatal care were the factors that contributed most to the observed greater number of cesareans in 2004. The authors admitted that the increase in the number of cesarean sections for non-medical reasons suggests a permeability in culture for cesarean births from the private to the public system, but do not indicate the pathways for this occurrence^12^. One possible mechanism would be a negotiation between pregnant women and physicians. It has been suggested that pregnant women with a higher socioeconomic status could increase their chance of having a cesarean section through prenatal appointments or “extra or illegal” payments for their physicians. In this scenario, they would be scheduled a medical appointment in their private clinics or define this payment by direct negotiation^15^. Nevertheless, this mechanism seems to be very unlikely in the three hospitals included in our study because of their teaching and research affiliations. Moreover, this type of agreement between patients and doctors is illegal and susceptible to lawsuits.

Similarly, in 2007, a study conducted in Rio Grande do Sul compared cesarean section rates between public and private services. Results show that higher education and income were associated with cesarean section in the public sector. The authors believe that maternal preference and medical practice may explain this association, but it is unclear whether this refers only to private services and how it happens in the public service^17^. On the other hand, a case-control study with 631 pregnant women conducted in a public maternity hospital in Rio de Janeiro did not observe an association between higher maternal education and cesarean section. However, it is unclear whether the teams had adopted standardized obstetric protocols and if these protocols were more flexible, since the authors reported an association between cesarean sections and maternal request^6^.

In disagreement with our results, Barros et al.^2^ evaluated 4,126 births in private and public sector, during the year of 2004, in Pelotas, RS, Southern Brazil. The authors found that cesarean sections were positively associated with maternal education level only among public patients. They considered that more educated mothers can be more persuasive to receive a cesarean section or that obstetricians perceive these women as closer to private patients and thus deserving a cesarean delivery. Alternatively, physicians may opt for a cesarean section to avoid the risk of lawsuits. In our study, the three public hospitals do not allow cesarean upon maternal request and the obstetric care is mostly offered by an obstetric team.

Some limitations of this study should be considered. First, there is the possibility of non-differential misclassification of socioeconomic status variables. However, to minimize this risk, three explanatory variables were used and none of them was associated with cesarean delivery in the adjusted model. Regarding the risk of non-differential misclassification due to the error in the assessment of obstetric variables, we should mention that data from medical records are relatively objective (e.g. low birth weight). Moreover, these hospitals have a great concern with the quality of data in the medical charts. However, non-differential misclassification would result in reduction of association between sociodemographic indicators and cesarean section. Second, the lack of association between the explanatory variables and cesarean section may be due to over-adjustment of the final model. However, we consider this final adjustment necessary to avoid residual confounding. Also, only the residential density variable lost significance after this adjustment, and the other variables, monthly family income and years of education, had lost significance previously (after adjustment in the model 2). Although the exposure variables used measure different aspects of the socioeconomic status, the lack of association of cesarean delivery with the three variables seems more logical. Although possible, it would be difficult to explain the presence of association with one indicator, but not the other. Third, since the data were collected in 2005, we are unable to state whether there is a change in the association between indicators of socioeconomic status and cesarean in these public hospitals. Nevertheless, the hospitals aforementioned still use standardized protocols of obstetric care. Finally, considering the type of maternity evaluated in this study (teaching public and private hospital, both with standardized protocols for obstetric care) and the sample size, our results cannot be generalized to other public services of the Country, particularly for those that do not adopt well-defined obstetric protocols.

The strength of this study includes the size of the sample, consisting of 831 pregnant women in low and lower-middleclass, attending public services. Further, we gathered data from 757 births for this analysis, which means a follow-up rate of 91.0%.

The increased caesarean section rates in Brazil in the private sector and, more recently, in the public sector cause concern because of the risks associated with this procedure. However, the effectiveness of measures to reduce cesarean section rates in private and public sectors is uncertain. The use of second opinion to perform a cesarean section^1^, restructuring of medical education, and changes in the way physician’s and hospital’s payment are performed are complex attempts and difficult to implement. Evidence suggests that interventions that emphasize the education of obstetricians and patients about the risk factors for the birth process are insufficient^8^. On the other hand, in the private sector (and possibly also in some public services), it becomes difficult to limit the autonomy of women, especially those with higher income, and obstetricians, to demand or to suggest cesarean, respectively. In this sense, the adoption of standardized obstetric protocols ensures greater equity in the care of women undergoing delivery.

## Footnotes

a Ministry of Health (BR), DATASUS, Inter-Agency Health Information Network. Basic Indicators for Health in Brazil – IDB 2012: F.8 proportion of cesarean deliveries [cited 2014 Oct 27]. Available from: http://tabnet.datasus.gov.br/cgi/deftohtm.exe?idb2010/f08.def

b National Agency of Health (BR). Brazil has one of the highest rates of caesarean section in the Supplementary Health. Brasília (DF) [cited 2015 Oct 15]. Available from: www.ans.gov.br/portal/site/home2/destaque_22585_2.asp

c Ministério da Saúde (BR), Secretaria de Vigilância em Saúde, Departamento de Análise de Situação em Saúde. Saúde Brasil 2012: uma análise da situação de saúde e dos 40 anos do Programa Nacional de Imunizações. 1. ed. rev. Brasília (DF); 2014 [cited 2015 Oct 15]. Available from: http://bvsms.saude.gov.br/bvs/publicacoes/saude_brasil_2012.pdf

d Ministry of Health (BR). Department of Health Policy, Program for Humanization of Prenatal and Birth. Brasília (DF); 2000.
